# Neutralizing monoclonal antibodies as effective therapeutics and prophylactics against lethal H10N7 avian influenza infection in a mouse model

**DOI:** 10.1186/s13567-025-01504-0

**Published:** 2025-04-02

**Authors:** Ping Wang, Jiamin Fu, Linfang Cheng, Sijing Yan, Han Wu, Fumin Liu, Hangping Yao, Nanping Wu, Lihua Xu, Haibo Wu

**Affiliations:** 1 State Key Laboratory for Diagnosis and Treatment of Infectious Diseases, and National Clinical Research Center for Infectious Diseases, School of Medicine, The First Affiliated Hospital, Zhejiang University, Hangzhou, 310003 China; 2 Animal Husbandry and Veterinary Institute, Zhejiang Academy of Agricultural Science, Hangzhou, 310021 China

**Keywords:** Avian influenza virus, H10 subtype, monoclonal antibodies, neutralizing, hemagglutination

## Abstract

The H10 subtype of avian influenza virus (AIV) is widespread in poultry worldwide and poses a significant threat to animal health. With the emergence of sporadic and fatal cases in humans infected with H10 subtype AIVs in recent years, it is imperative to develop neutralizing monoclonal antibodies (mAbs) to treat influenza clinically. In this study, BALB/c mice were immunized with A/chicken/Zhejiang/2CP8/2014 (H10N7) haemagglutinin (HA) protein, and eight HA-specific mAbs were subsequently screened. The characteristics of the mAbs were tested and evaluated using haemagglutination inhibition and microneutralization assays in vitro. We selected two mAbs (1E10 and 2A9) to further study their characteristics and functions, including their affinity and specificity of binding to antigens via enzyme-linked immunosorbent assays and immunofluorescence assays. We identified the mutant epitopes (K165E and N170D) of the H10N7 strain produced under the immune pressure of the two mAbs. Furthermore, we infected mice with the H10N7 virus and conducted prophylactic and therapeutic trials using the two mAbs. The results indicated that both mAbs have obvious neutralization ability in vivo. Compared with those in the isotype IgG control group, the weights of the mice in the experimental groups were greater in the prophylactic and therapeutic experiments. In conclusion, the mAbs produced in this study are expected to be effective drugs for clinical antiviral therapy against lethal infection by H10 AIVs.

**Supplementary Information:**

The online version contains supplementary material available at 10.1186/s13567-025-01504-0.

## Introduction

Avian influenza viruses (AIVs) have negative-sense, single-stranded, and segmented RNA genomes and are classified within the *Orthomyxoviridae* family. The viral envelope is characterized by the presence of two key glycoproteins: haemagglutinin (HA) and neuraminidase (NA) [1]. AIVs, such as H7N9 and H5N1 AIVs, are widely prevalent in live poultry markets, pose a significant threat to the health of both humans and birds, and have substantial impacts on social stability and economic development [2–4].

In March 2010, H10N7 AIV infection occurred in a commercial poultry enterprise in Australia and caused sporadic human infections with clinical symptoms [5]. Typical clinical symptoms include high fever, chills, headache, and respiratory symptoms, such as cough, phlegm, and sore throat. Pneumonia is considered the most serious complication of influenza, especially in elderly individuals and patients with immune dysfunction [6, 7]. In 2014, there was an outbreak of H10N7 AIV in seal populations in northern European coastal waters, which suggested cross-species transmission and mammalian adaptation of H10N7 AIV infection [8, 9]. In recent years, there have been frequent sporadic and fatal cases of H10 AIV infections in humans in China. In 2013, there were three human cases of infection with the H10N8 strain in Jiangxi, China, 2013, two of which were fatal. Additionally, in 2021, the first human infection with the H10N3 AIV strain occurred in Jiangsu Province, China [10, 11]. In 2023, an H10N5 strain was isolated in Zhejiang Province, China, which was coinfected with H3N2 and led to patient death [12, 13]. Considering the current prevalence of H10 AIVs, strengthening research on treatment to address potential outbreaks of infection is essential.

Currently, antiviral drugs and antibodies are the principal treatments for influenza virus infection. However, the production of vaccines is time-consuming and may not necessarily match the current epidemic strain [14]. Moreover, antiviral drugs, such as NA inhibitors (Oseltamivir, Zanamivir, and Peramivir) and a polymerase acidic (PA) protein (baloxavir), cannot completely cure influenza infection and can shorten the duration of clinical remission [15]. Moreover, as drug-resistant strains emerge, antiviral drugs become ineffective [16]. Monoclonal antibodies (mAbs) are used as preventive and therapeutic drugs for viral infection because of their high specificity and ability to enhance immune responses [17]. Current analytical studies have demonstrated that monoclonal antibody drugs such as motavizumab, nirsevimab, and palivizumab have significant effects on preventing respiratory syncytial virus (RSV) infections in infants and children [18, 19]. Broadly neutralizing mAbs against HIV-1 envelope proteins can block the acquisition of the virus and even increase the body’s immune capacity [20]. Additionally, clinical trials have confirmed that the safety and tolerability of HIV-1 glycan-specific mAbs are satisfactory [21]. In recent coronavirus disease 2019 (COVID-19) outbreaks, a mAb against the severe acute respiratory syndrome coronavirus 2 (SARS-CoV-2) spike protein was one of the main therapeutic drugs used to prevent infection-related lung diseases through early intervention [22]. MAbs against HA and NA glycoproteins are pivotal for continuously suppressing prevalent influenza viruses, which bind to influenza viruses and robustly inhibit the activity of HA and NA enzymes, thereby significantly blocking influenza virus infection and preventing infected cells from releasing progeny viruses [23–25].

In this study, we produced eight HA-specific mAbs against H10 subtype AIVs and evaluated their characteristics and functions. The results showed that these mAbs have a broad-spectrum neutralization effect. We selected two mAbs (1E10 and 2A9) for further functional characterization, including their affinity and specificity for binding with antigens. We infected mice with the H10N7 virus and conducted prophylactic and therapeutic trials with the two mAbs, which showed an ideal antiviral effect in these mice.

## Materials and methods

### Ethics approval

All animal experiments were performed according to the protocol of the Animal Ethics Procedures and Guidelines of the People’s Republic of China, and the study was approved by the Animal Ethics Committee of the First Affiliated Hospital, School of Medicine, Zhejiang University (No. 2023-1033). Experiments involving highly pathogenic influenza viruses were conducted in biosafety level 3 laboratories, strictly following the biosafety management manual.

### Cells and viruses

The Madin‒Darby canine kidney (MDCK) cell line was obtained from the American Type Culture Collection (Rockville, MD, USA) and cultured in Dulbecco’s modified Eagle’s medium (complete DMEM, Gibco, Grand Island, NY, USA), which was supplemented with 10% foetal bovine serum (FBS, Gibco) and 1% antibiotic solution (penicillin [100 U/mL] and streptomycin [100 U/mL], Pen-Strep, Gibco). Many strains of the virus are stored in our laboratory (Table 1). All the viruses were amplified in 9-day specific pathogen-free (SPF) embryonated chicken eggs. The viruses were subsequently titrated by a standard tissue culture infectious dose 50 (TCID_50_) test and stored at −80 °C.

**Table 1:** **The virus strains used in this study**

Virus strains	Subtype
A/duck/Zhejiang/6D20/2013 (H10N2)	H10N2
A/chicken/Zhejiang/8615/2016 (H10N3)	H10N3
A/Zhejiang/CNIC-JU01/2023 (H10N5)	H10N5
A/chicken/Zhejiang/2CP8/2014 (H10N7)	H10N7
A/chicken/Zhejiang/121711/2016 (H10N8)	H10N8
A/Michigan/45/2015 (H1N1)	H1N1
A/duck/Zhejiang/6D10/2013 (H2N8)	H2N8
A/duck/Zhejiang/4613/2013 (H3N2)	H3N2
A/duck/Zhejiang/727145/ 2014 (H4N2)	H4N2
A/goose/Zhejiang/112071/2014 (H5N1)	H5N1
A/chicken/Zhejiang/1664/2017 (H6N1)	H6N1
A/chicken/Zhejiang/DTID-ZJU01/2013 (H7N9)	H7N9
A/chicken/Zhejiang/329/2011 (H9N2)	H9N2
A/duck/Zhejiang/727D2/2013 (H11N3)	H11N3

### Production of monoclonal antibodies

Five female BALB/c mice (6 weeks old, Shanghai Laboratory Animal Centre) were intramuscularly injected with purified H10N7 HA protein, and a Quick Antibody adjuvant (Biodragon, Beijing, China) was added to increase immunogenicity. Three weeks later, the same immunization was administered so that the mice underwent a primary and secondary immune response. The antibody levels in the tail vein serum of the mice were detected by enzyme-linked immunosorbent assay (ELISA). Splenocytes were extracted from mice with higher antibody levels and then fused with SP2/0 myeloma cells [26]. Three days before fusion, the mice received an additional intraperitoneal injection of the same HA protein without adjuvant. Approximately every other week, the antibody dose in the hybridoma culture supernatants was monitored using ELISA. The positive hybridoma cell lines were cloned using limited dilution. Finally, the hybridoma lines determined to be monoclonal at least three times in a row with positive results were selected for proliferation culture. When the hybridoma cell clones were fully expanded, the cells were collected and intraperitoneally injected into BALB/c mice. After approximately ten days, the ascites of the mice were obtained and purified using a protein-G column (GE Healthcare, Chicago, IL, USA). The concentrations of the purified mAbs were detected using a Nanodrop 2000 spectrophotometer (Thermo Fisher Scientific, Waltham, MA, USA). The antibodies were stored at −80 °C. A mouse monoclonal antibody isotyping kit (Bio-Rad, Hercules, CA, USA) was then used to determine the subclass of the mAbs. The heavy- and light-chain genes of the hybridoma cells were subsequently sequenced.

### ELISA

H10N7 HA protein was coated on the wells of 96-well plates overnight at 4 °C. The next day, 100 μL of individual hybridoma culture supernatant was added to each well, followed by incubation with a goat anti-mouse IgG secondary antibody (horseradish peroxidase [HRP]-linked; Novus Biologicals, Toronto, Canada). Next, 3,3′,5,5′-tetramethylbenzidine (TMB) staining solution was added, followed by stop solution. The optical density (OD) was then measured at 450 nm on a SpectraMax Absorbance Reader (Molecular Devices, San Jose, CA, USA). The affinity of the mAbs was evaluated using ELISA as described previously [27]. MAbs at an initial concentration of 0.1 μg/mL were continuously diluted twofold in phosphate-buffered saline (PBS) and then detected by ELISA.

### Haemagglutination inhibition (HI) assay

Before the HI assay, the viruses were titrated with 1% chicken red blood cells. The mAbs were then serially diluted twofold in PBS and incubated with the same amounts of target viruses (4 HA units) for 30 min in V-bottomed 96-well microtiter plates at room temperature. Finally, 1% chicken erythrocytes were added to each well and incubated at room temperature for 30 min. The concentration of the mAbs that suppressed haemagglutination after the highest dilution was identified as the HI titre.

### In vitro microneutralization (MN) assay

The mAbs were diluted to the appropriate concentration and subsequently diluted twofold in 96-well plates. The mAbs were subsequently mixed with an equal volume of 100 TCID_50_ H10 virus per well. The mixture was incubated at 37 °C in a culture incubator for 2 h. The supernatant of the MDCK cells that were pre-grown at a density of 2 × 10^4^ per well in 96-well plates and showed exponential growth was discarded. After the MDCK cells were washed with PBS, the antibody and virus mixture were transferred to 96-well plates. The concentration of mAbs that could reduce virus replication by 50% was evaluated using the HI assay, and the neutralizing efficacy of the mAbs was determined according to the Reed-Muench method [28].

### Immunofluorescence (IFA)

MDCK cells were inoculated into 48-well plates at a density of 2 × 10^4^ cells per well in advance. Different types of target viruses were diluted appropriately to achieve a multiplicity of infection (MOI) of 0.5 for infection of the MDCK cells, followed by culture in an incubator at 37 °C for 16 h. After the cells were fully infected, the cells were fixed and permeabilized at room temperature with 4% paraformaldehyde and 0.5% Triton-X100 for 30 min each and then blocked with 3% bovine serum albumin (BSA) at room temperature for 1 h. The target antibody was diluted to 10 μg/mL with PBS and incubated with the cells at 4 °C overnight. The next day, goat anti-mouse IgG-Alexa Fluor 488 (Abcam) diluted to 5 μg/mL with 1% BSA solution was added, and the mixture was incubated at 37 °C for 90 min in the dark. Thereafter, the nuclei of the MDCK cells were stained with 4′,6-diamidino-2-phenylindole (DAPI) for 10 min at room temperature without light. The MDCK cells were washed 1–3 times with PBS before the above reagents were added. Finally, we observed the staining results under a fluorescence microscope after the MDCK cells were rinsed with PBS three times.

### Immune escape mutation

The mAbs were diluted to 2 and 4 ng/mL in PBS and mixed with the same volume of 100 TCID_50_ of H10N7 influenza virus (A/chicken/Zhejiang/2CP8/2014). After the diluted mAbs and virus were mixed thoroughly, the mixture was inoculated into 9-day-old chicken embryos and incubated for 48 h at 35 °C. Allantoic fluid was then harvested, and viral activity was assessed using the HA assay. The concentrations of the mAbs were then increased to 8 and 16 ng/mL. The mAbs were added to the positive allantoic fluid, and the inoculation and cultivation processes were repeated. After the last round of mutation selection, the HA-positive allantoic fluid was collected to extract viral RNA. The viral gene sequences were determined, aligned, and compared with those of the original strain to determine whether mutants were produced and to identify the corresponding amino acid substitutions [26].

### Prophylactic and therapeutic effects

Sixteen seven-week-old female BALB/c mice were intranasally infected with 50 μL of H10N7 influenza virus (A/chicken/Zhejiang/2CP8/2014) to identify the 50% mouse lethal dose (MLD_50_). In the prophylactic experiment, four groups of mice (*n* = 10 per group) were intraperitoneally injected with different doses of each antibody (0.3, 1, 3 and 10 mg/kg), and the mice in the control IgG group were administered 10 mg/kg of a mouse isotype IgG (Solarbio, Beijing, China). Each group of mice was given 200 μL of the corresponding antibody 6 h before infection with the virus (at 5 times the MLD_50_). To study the therapeutic efficacy of the mAbs, eight groups of mice (*n* = 10 per group) were intraperitoneally injected with different doses of each antibody (0.3, 1, 3, and 10 mg/kg) along with a control IgG group at 12 and 24 h post-infection. Three mice in each group were euthanized on days 3 and 6. Their lung tissues were excised, and one half of the lung tissues were preserved in a fixative solution for sectioning of pathological tissue samples and analysis of their histopathological characteristics. The other half was homogenized in PBS to determine the pulmonary virus titre by the TCID_50_ assay.

### Paraffin sectioning and haematoxylin–eosin (H&E) staining

Fresh mouse lung tissues were stored in fixative solution (10% formaldehyde and Bouin solution) at room temperature for 4 to 24 h and then dehydrated in low to high concentrations of alcohol. The tissues were then immersed in xylene for 5–30 min, immediately immersed in melted paraffin wax for 2–3 h, and then placed in an embedding tank with melted paraffin wax. After the paraffin wax solidified, the tissues were sectioned into thin slices. The sections were then dewaxed and hydrated sequentially with xylene, alcohol, and distilled water. The nuclei were stained with haematoxylin, and the cytoplasm was stained with eosin. The sections were mounted and observed under a microscope [29].

## Results

### HI and MN activities of eight mAbs against H10 virus in vitro

To identify whether the eight mAbs could act on the HA protein of H10 viruses, we evaluated the HI activity of the mAbs against five types of H10 influenza viruses and other influenza viruses (Table 1). As shown in Table 2, mAbs 1E8 and 1E10 presented the strongest HI activity against H10N2 and H10N3, at 0.39 and 0.78 μg/mL, respectively. The HI capacity of mAbs 2D8, 2E9, and 2A9 against H10N2 and H10N3 was relatively weak, whereas the HI concentration against H10N7 of mAb 2A9 was as low as 0.09 μg/mL, which was significantly lower than that of the other mAbs. In the HI assay against H10N8, mAbs 1E10 and 2A9 exhibited the most significant ability to inhibit chicken erythrocyte agglutination, followed by 1E8, 1E1, and 2E8, with only a one-fold difference between them. In general, the HI ability of these eight mAbs against H10N5 was weaker than that against the other H10 subtypes, and the concentration of mAb 2A9 that inhibited H10N5 was also the lowest, reaching 7.81 μg/mL. Furthermore, the eight mAbs all showed HI reactivity against H10 subtype influenza virus, which suggested that they all have the potential to neutralize H10 subtype influenza viruses.

**Table 2:**
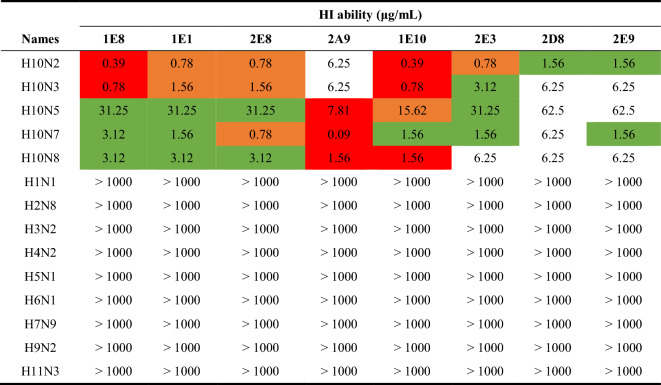
**The HI abilities of eight mAbs against influenza viruses**

The HI ability, referred to as the 50% inhibition concentration (IC_50_), was measured by the HI assay against H10 and other subtype influenza viruses. The initial concentration of the mAbs was 100 μg/mL, and the lowest concentration at which haemagglutination was completely inhibited was defined as the HI ability. For HI ability against H10N2, H10N3, H10N7, and H10N8, values greater than 6.25 μg/mL, and for HI ability against H10N5, values greater than 62.5 μg/mL are marked in red for high capacity, orange for moderate-high capacity, and green for moderate capacity.

**Figure 1:**
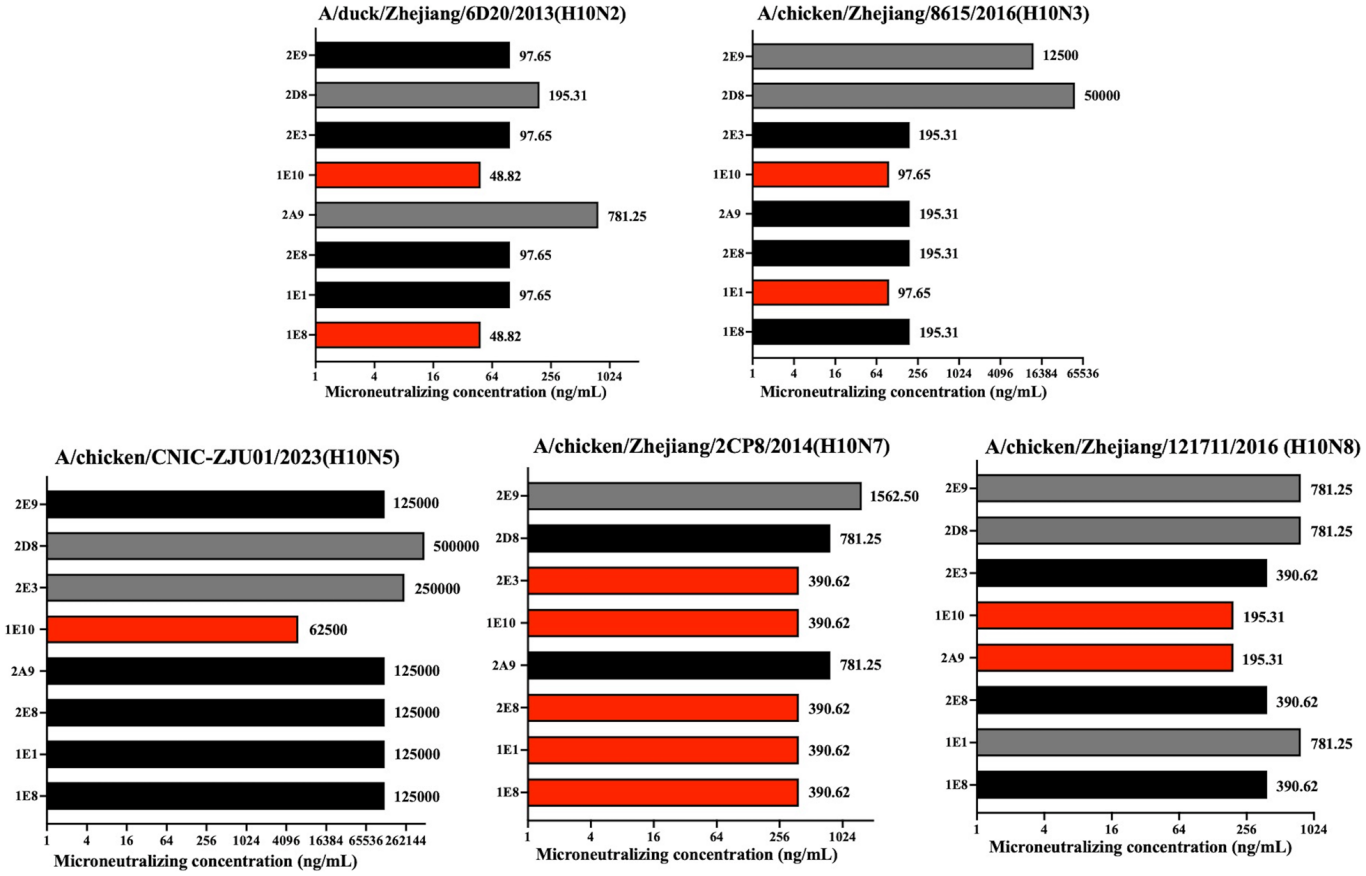
**The microneutralization concentration (IC**_**50**_**) against H10 subtype influenza viruses**. The lowest concentration of neutralization was defined as the MN ability. The initial concentration of antibodies was 100 μg/mL, while the initial concentration of antibodies used to neutralize H10N5 was 500 μg/mL. The above statistics are represented by average values of three independent in vitro microneutralization experiments. The columns in red, black, grey and white represent strong, moderately strong, medium, and medium or weak neutralizing reactivity, respectively.

To determine the in vitro microneutralizing ability of the mAbs, we also conducted MN assays with the mAbs against the five types of H10 subtype influenza virus mentioned above. The experiments revealed that the eight mAbs all had neutralization activity against the five types of H10 subtype influenza viruses (Figure 1). Interestingly, in general, they had the strongest neutralizing effect against H10N2 but the weakest effect against H10N5. On the basis of the experimental data, mAb 1E10 demonstrated the strongest neutralization ability against all five H10 subtype influenza viruses, with a minimum effective concentration that was significantly lower than that of the other mAbs. Specifically, the minimum neutralizing concentrations of 1E10 against H10N2, H10N3, H10N5, H10N7 and H10N8 were 48.82, 97.65, 6250, 390.62, and 195.31 ng/mL, respectively. In neutralization experiments involving the H10N7 strain, the mAbs 1E10, 1E8, 1E1, 2E8, and 2E3 demonstrated favourable reactivity. Under the same conditions, the neutralizing ability of mAb 2A9 was the strongest against H10N8, the same as that of 1E10, with a minimum concentration of 195.31 ng/mL. Overall, these results indicated that the 1E10 antibody has an ideal neutralizing effect against H10 subtype viruses under the experimental conditions. For use in the H10N7-infected mouse model, we ultimately selected mAbs 1E10 and 2A9, which have ideal HI reactivity and microneutralization ability, for further experiments.

### Binding of 1E10 and 2A9 to HA of the H10N7 virus

**Figure 2:**
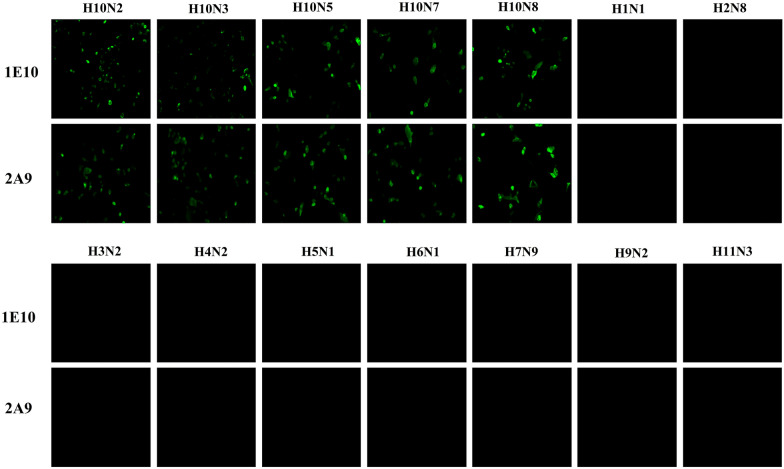
**Immunofluorescence assay to evaluate the specificity of the binding of mAbs 1E10 and 2A9 to influenza viruses**. After MDCK cells were infected with influenza viruses (H10N2, H10N3, H10N5, H10N7, H10N8, H1N1, H2N8, H3N2, H4N2, H5N1, H6N1, H7N9, H9N2, and H11N3), they were fixed, permeated, and blocked. The cells were then incubated with mAbs 1E10 and 2A9 and stained with goat anti-mouse IgG-Alexa Fluor 488-conjugated secondary antibodies (green) and DAPI, successively. Images were captured using fluorescence microscopy.

We selected two mAbs (1E10 and 2A9), and their hybridoma culture supernatants were used for isotype detection, as shown in Table 3. MAbs 1E10 and 2A9 belong to the IgG2b and IgG2a isotypes, respectively. Both antibodies strongly bound to the antigen (the H10N7 HA protein). As shown in Table 3, their affinity values reached 2 × 10^–4^ and 4 × 10^–4^ μg/mL, respectively. The sequencing results of the monoclonal hybridoma cell line revealed that the heavy and light chain gene sequences of the two mAbs were different. Next, we evaluated the specificity of the mAbs for binding to various influenza viruses using IFA. The results showed that 1E10 and 2A9 could specifically bind to H10 subtype influenza viruses but could not bind to other subtype influenza viruses (Figure 2).

**Table 3:** **Characteristics of mAbs 1E10 and 2A9 recognizing H10 influenza viruses**

mAbs	Isotype	Affinity (μg/mL)	Heavy chain sequence		Light chain sequence	
			V-gene	CDR3	V-gene	CDR3
1E10	IgG2b,k	4 × 10^–4^	I GHV8-12*02 F	ARSPPTDYGSSWGVMDY	I GKV4-59*01	QQWSSNPL
2A9	IgG2a,k	2 × 10^–4^	I GHV9-3-1*01 F	ARGYDYAEGYFAMDY	I GKV8-30*01 F	QQYYSNYT

The isotypes of the monoclonal antibodies were determined using a mouse monoclonal antibody isotyping kit. The affinity of the mAbs was evaluated using ELISA as described in the materials and methods section. The V-gene is the DNA sequence encoding the variable region (region V). CDR3 indicates complementarity-determining region 3.

### Mutant epitope identification by selection of 1E10 and 2A9

**Figure 3:**
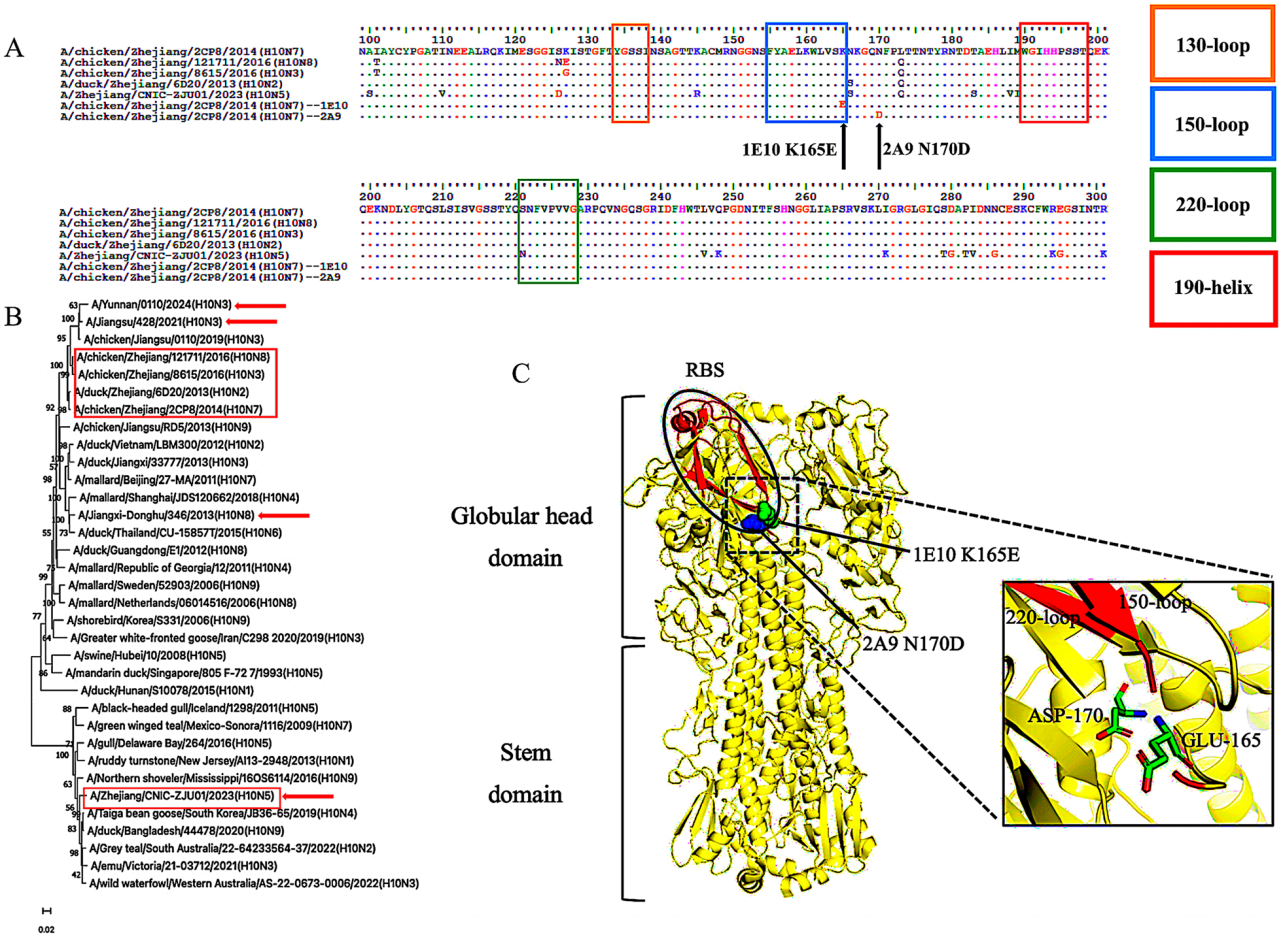
**Phylogenetic analysis and mutation epitope simulation**. **A** Sequence alignment analysis results of H10N7 subtype influenza virus (A/chicken/Zhejiang/2CP8/2014) wild-type and mutant viruses under 1E10 and 2A9 antibody pressures, as assessed using BioEdit. The 130-loop, 150-loop, 220-loop, and 190-helix regions are marked with yellow, blue, green, and red frames, respectively. The black arrow indicates the site of amino acid substitutions. **B** Phylogenetic analysis of HA genes of H10 subtype virus strains displayed in an evolutionary tree. Information on the HA genes of representative H10 subtype viruses was downloaded from the National Centre for Biotechnology information database and GISAID. MEGA11 was used to analyse the gene sequences to obtain the evolutionary tree. The H10 subtype strains used in this experiment are marked with red frames. The red arrow points to the H10 strains that have infected humans. **C** Simulation of the three-dimensional crystal structure and mutant epitopes of the H10 subtype virus created using PyMoL 3.1. The crystal structure information was downloaded from the Protein Data Bank (PDB: 6TJW). The N170D mutant epitope of 2A9 is shown as a blue sphere, whereas the K165E mutant epitope of 1E10 is shown as a green sphere. The circled part refers to the receptor binding site (RBS). The figure on the right shows the two mutated amino acids in a ribbon form, with the C, H, N, and O atoms marked in green, yellow, blue, and red, respectively.

By inoculating a mixture of the two mAbs and H10N7 virus into chicken embryos and increasing the concentrations of the mAbs at each round of inoculation, the viral gene sequences were mutated under the immune pressure of the mAbs, allowing identification of the active epitope. Through experiments and comparisons of the virus sequences, we detected that an amino acid substitution at HA residue 165 (K165E) was affected by 1E10, whereas 2A9 treatment contributed to an N170D mutation (Figure 3A). The results of the polymorphism analysis suggested that the K165 and N170 sites are conserved among 1837 H10 HA proteins, with percentages greater than 99%. In addition, the probabilities of lysine (K) mutation at site 165 to glutamate (E) and asparagine mutation at site 170 to aspartic acid were very low, at 0.22% and 0.11%, respectively (Table 4). The K165E substitution was observed in four strains: A/duck/Manitoba/1953 (H10N7), A/duck/Hubei/137/1985 (H10N4), A/duck/Hong Kong/934/1980 (H10N5), and A/ruddy turnstone/King Island/14063/2019 (H10N5), whereas the N170D substitution was observed in two strains: A/duck/Hokkaido/18/2000 (H10N4) and A/chicken/Zhejiang/HY25/2021 (H10N3).

**Table 4:** **Polymorphism analysis of mutant residues in H10 subtype influenza viruses**

MAbs	Residues	Percentage of amino acid substitution sites in H10 strains
1E10	K165E	K (99.40%, 1826/1837), G (0.27%, 5/1837), E (0.21%, 4/1837), N (0.05%, 1/1837), R (0.05%, 1/1837)
2A9	N170D	N (99.45%, 1827/1837), T (0.21%, 4/1837), S (0.16%, 3/1837), D (0.10%, 2/1837), K (0.05%, 1/1837)

A total of 1837 H10 HA gene sequences up to 27 November 2024 from the GISAID were analysed and aligned using BioEdit. The percentages in the table represent the proportion of each amino acid at positions 165 and 170 in the circulating H10 strains.

By simulating the three-dimensional crystal structure of the H10 virus, it was observed that the 165th site was located in the 150-loop region, whereas the mutation at site 170 was located near the 150-loop region, and both are close to the 220-loop region and closely related to the receptor binding site (RBS) (Figure 3C).

Furthermore, a comparison of the gene sequences of H10 virus strains isolated from different regions and from different years revealed that the H10 virus strain used in this study is highly similar to the H10N3 virus strains A/chicken/Jiangsu/428/2021 and A/Yunnan/0110/2024, which infect humans in Jiangsu and Yunnan Provinces, China, in 2021 and 2024 [30, 31]. Moreover, the H10N5 virus strain (A/Zhejiang/CNIC-ZJU01/2023) we used is actually the strain that coinfected humans with H3N2 and led to death in 2023 [13] (Figure 3B).

### Protective effects of the mAbs in vivo

**Figure 4:**
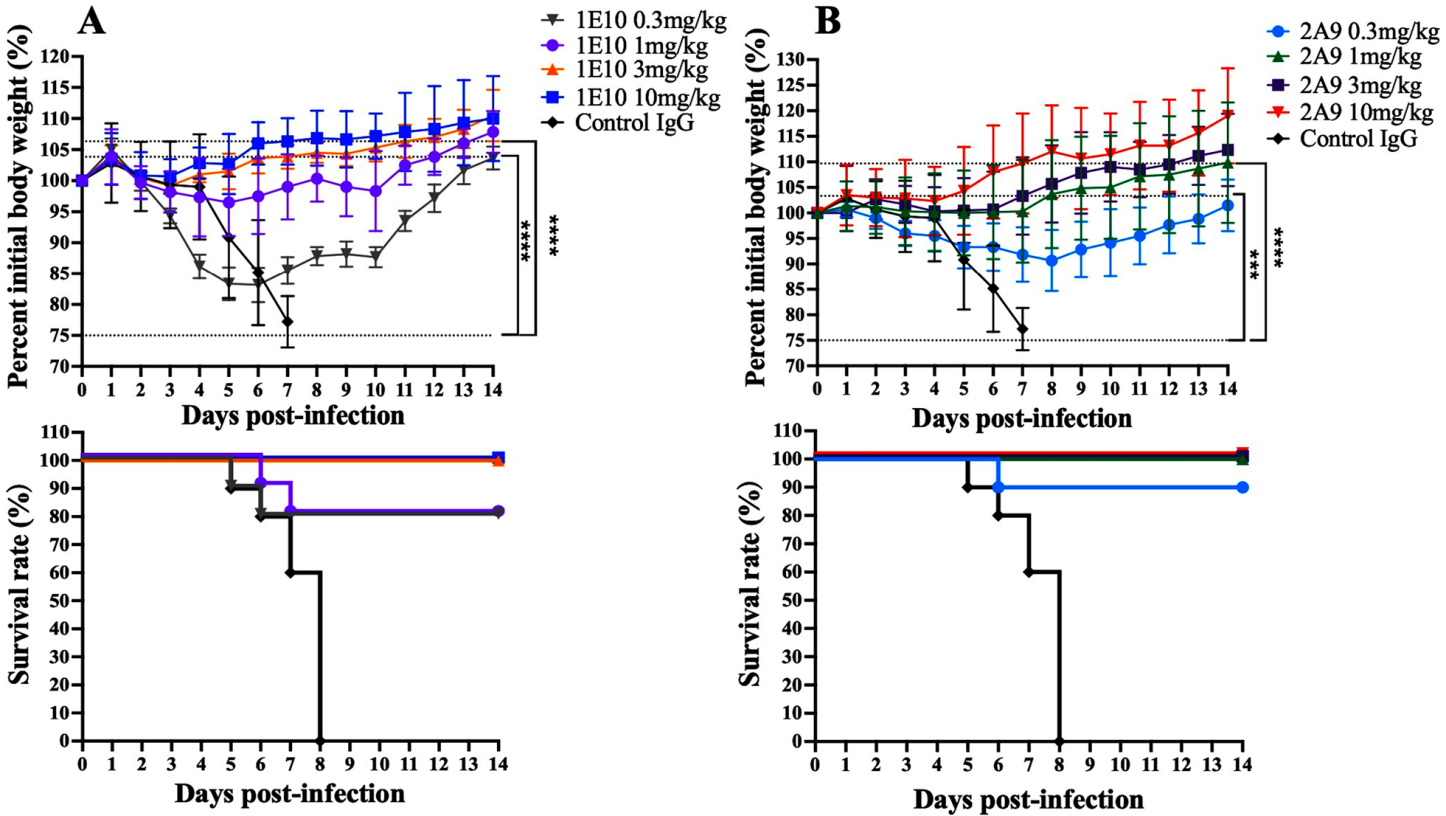
**Prophylactic effects of the 1E10 and 2A9 mAbs in mice.** The body weight curves and survival curves of the 1E10 (**A**)- and 2A9 (**B**)-treated groups of mice (*n* = 10 per group). The mice in the experimental groups were injected with the corresponding concentrations of antibody (0.3, 1, 3, or 10 mg/kg) 6 h before being infected with 5 × the MLD_50_ of H10N7 virus (A/chicken/Zhejiang/2CP8/2014), whereas those in the control group were injected with 10 mg/kg of isotope IgG. Differences in average weight between the mAb groups and the control IgG group on day 7 post-infection were determined by two-way analysis of variance (ANOVA). Groups with a *p* value less than 0.001 (*** < 0.001, **** < 0.0001) are indicated on the image. The above images were obtained using GraphPad Prism 10.

To identify whether 1E10 and 2A9 have in vivo neutralizing effects, we infected mice with the H10N7 virus and conducted prophylactic and therapeutic trials. The results revealed that the protective effects were enhanced with increasing doses of 1E10 and 2A9 (Figure 4). Prophylactic research revealed that doses of 3 mg/kg and 10 mg/kg 1E10 provided 100% protection, with a survival rate of 100% in the mice and no significant weight loss within two weeks. Moreover, the protection rate of 1E10 at doses of 0.3 and 1 mg/kg in mice was 80%. The average weight in the low-dose groups was generally lower than that in the high-dose groups. Overall, the weight of the mice that received 1 mg/kg of 1E10 clearly fluctuated, whereas the weight of the mice that received 0.3 mg/kg of 1E10 increased earlier but began to recover on day 6. In addition, the survival rates of the 2A9 group of mice at doses of 1, 3, and 10 mg/kg were 100%, while 0.3 mg/kg 2A9 provided 90% protection, with a more significant weight increase trend than that of the mice injected with 1E10. The average weights of the mice in the 1E10 and 2A9 experimental groups were greater than those in the IgG control group (Figure 4).

**Figure 5:**
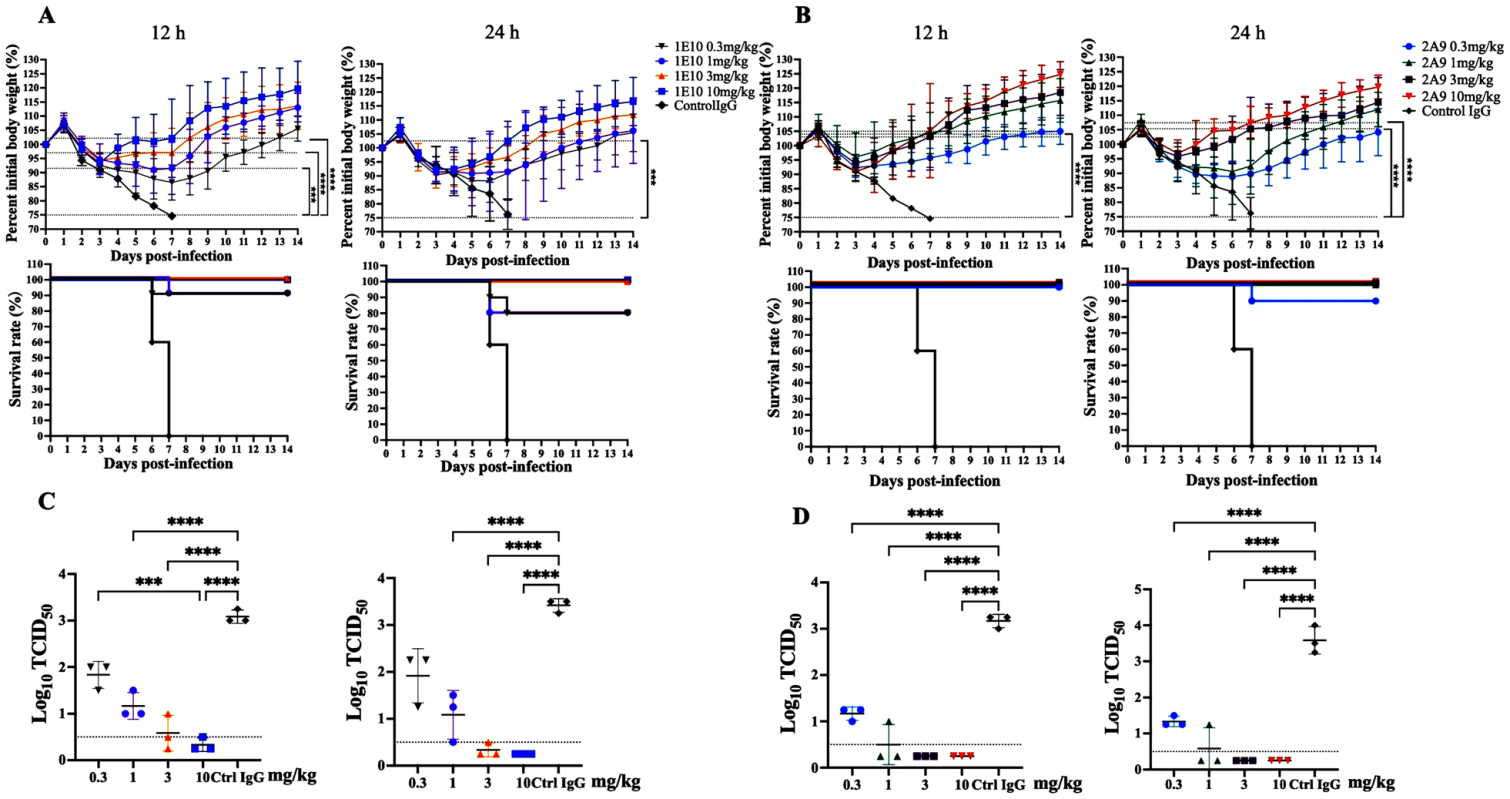
**Therapeutic effects of 1E10 and 2A9 in mice and the virus load in mouse lung tissues.** The body weight curves and survival curves of mice treated with various concentrations (0.3, 1, 3, and 10 mg/kg) of 1E10 (**A**) or 2A9 (**B**) and infected with 5 × the MLD_50_ of H10N7 virus (A/chicken/Zhejiang/2CP8/2014) at 12 and 24 h post-infection. Three mice in the 1E10 (**C**) and 2A9 (**D**) treatment groups were euthanized on days 3 and 6 to obtain the lung tissues, and the virus titres were measured via a TCID_50_ assay. Differences in average weight between the mAb groups and the control IgG group on day 7 post-infection and differences in virus titres between groups were determined by two-way analysis of variance (ANOVA). Groups with a *p* value less than 0.001 (*** < 0.001, **** < 0.0001) are indicated on the image. The above images were obtained using GraphPad Prism 10.

In the therapeutic trials, the protective effect of the mAbs on the mice was influenced not only by the dose but also by the timing of administration (Figure 5). We found that injecting 0.3 or 1 mg/kg of 1E10 at 24 h post-infection provided a protection rate of 80% for the mice. However, at the same dose, when 1E10 was injected at 12 h post-infection, the protection rate for the mice reached 90% (Figure 5A). Furthermore, the survival rate of the mice treated with mAb 2A9 was generally greater than that of the group treated with 1E10, and it also increased as the treatment time increased (Figure 5B). Compared with the control IgG, both antibodies had a 100% survival rate at a high dosage and prevented sustained weight loss in mice.

Additionally, through the determination of the viral load in lung tissues, we found that the viral load in the lungs of mice treated with the mAbs was lower than that in the lungs of the IgG control group (*p* < 0.5), and the viral loads in the 2A9 treatment groups and high-dose 1E10 treatment group were significantly lower than those in the IgG control group (*p* < 0.0001; Figures 5C and D). Compared with those in the control group, mice treated with low doses (0.3 and 1 mg/kg) of 1E10 presented a reduction in the mean viral load of approximately 40–65%, whereas mice treated with high doses (3 and 10 mg/kg) of 1E10 presented a reduction of 80–90%, which was nearly the same as that of the high-dose 2A9 group. However, the viral load in the lung tissues of mice given low doses of 2A9 was reduced by 60–90%. The data showed that the time of administration (12 or 24 h) after infection had no significant effect on the viral load.

**Figure 6:**
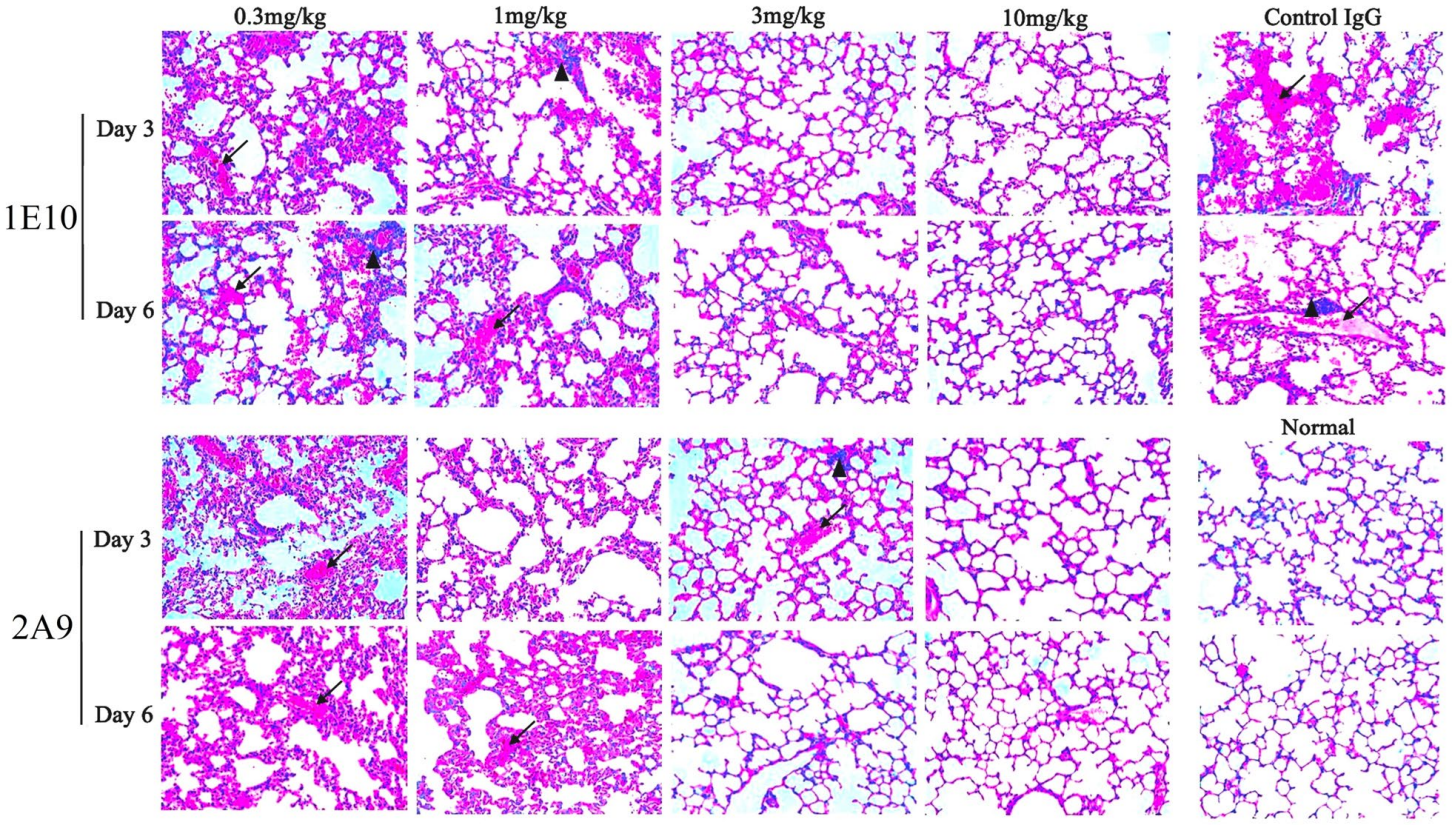
**Histopathological analysis of the lungs of mice in therapeutic experiments**. Paraffin sectioning and haematoxylin‒eosin (H&E) staining were performed. Three mice treated with various concentrations (0.3, 1, 3 and 10 mg/kg) of 1E10 or 2A9 and infected with H10N7 virus 24 h post-infection were euthanized on days 3 and 6 to obtain lung tissues. The arrows indicate the sites of congestion, erythrocyte accumulation, and interstitial fluid exudation between or within the alveoli, whereas the triangles mark the infiltration of immune cells, such as lymphocytes, in the interstitium and parenchyma of the lung tissues. Magnification = 40 ×.

By analysing lung histopathology changes, we observed that the control group mice exhibited multifocal interstitial inflammation and even diffuse alveolar damage after infection. Pulmonary interstitials, such as the bronchial epithelium and its surroundings, the lobular septum and the alveolar epithelium, showed congestion and oedema. There was also obvious infiltration of erythrocytes, lymphocytes, and basophil cells. Exudates composed of serous fluid, red blood cells, and some immune cells could be observed in the alveolae of the H&E sections. The pathological results of the lung tissues from the mice treated with the mAbs revealed that although there were different degrees of exudation of erythrocytes or inflammatory cells, such as lymphocytes, the overall degree of damage was mild, and there was no obvious pulmonary interstitial necrosis. Moreover, the lung tissues from the mice treated with higher antibody dosages presented the mildest lesions (Figure 6).

In summary, the 1E10 and 2A9 mAbs possess ideal neutralization effects in vivo and are capable of inhibiting viral replication, with 2A9 demonstrating a stronger protective effect than does 1E10.

## Discussion

The H10 subtype of influenza virus is distributed in both domestic and wild birds in China [32–35]. The H10 AIV is continuously circulating in China and has the potential for interpersonal transmission. The H10 AIVs used in this study have HA genes similar to those of the human H10 strains that have appeared in China in recent years, laying a partial foundation for addressing the possible emergence of new H10 strains in the future and facilitating research on other subtypes of AIVs.

Studies have verified the remarkable role of neutralizing antibodies in the treatment of influenza virus infection [36–38]. Previously, our research team obtained anti-H7 HA head antibodies against H7N9, which have desirable neutralizing effects, and confirmed their preventive and therapeutic effects in mouse infection models [39]. Other researchers have reported neutralizing antibodies against the most commonly circulating clades and subclades of H5 AIVs [40, 41]. Antibodies with broad-spectrum neutralizing effects hold greater clinical value. Indeed, the cross-neutralizing antibody C12H5 was reported to have protective effects against H5N1 and H1N1 viral challenge in mice [42]. Influenza B virus (IBV) can also result in an annual flu epidemic worldwide, and researchers have isolated a human mAb against IBV, 48B8, using a plasmablast enrichment technique. The results showed that this antibody had a stronger antiviral effect than Tamiflu, with the capacity to bind to a conserved epitope in the vestigial esterase domain of HA, thereby inhibiting membrane fusion [31]. On the basis of the above research, antibodies have been recognized as crucial means of clinical treatment for influenza and are a key focus of research in influenza prevention and control.

In this study, two mAbs, 1E10 and 2A9, showed broad-spectrum neutralizing capabilities against certain H10 subtype influenza viruses, including H10N2, H10N3, H10N5, H10N7, and H10N8, that circulate in China. In the HI assay, we observed that the mAbs bound to the HA glycoprotein on the surface of the viruses, which is the main target of neutralizing antibodies. The HA head domain mediates virus binding to the sialic acid receptors of host cells, whereas the stalk domain mediates membrane fusion between viruses and cells under low pH conditions in endosomes [43]. Recurrent infections with the human influenza virus occur predominantly because of antigenic drift of the HA protein [44]. A previous study tested H10N8 anti-HA head, anti-HA stalk, and anti-NA antibodies together and reported that anti-HA head-directed antibodies, which may keep NA away from its substrate, had greater in vitro neutralizing activity and showed superior protection against virus attack [45]. Two epitopes targeted by 1E10 and 2A9 include K165E and N170D, respectively, suggesting that their binding sites are located in the globular head domain of HA, which contains the sialic acid RBS, and antibodies against this region are typically specific to particular strains or even clades [43, 46]. Statistically, the two epitopes are highly conserved in most prevalent H10 influenza viruses. The conservation of the antigenic site accounts for the cross-reactivity observed with 1E10 and 2A9, allowing them to interact with H10 subtype influenza viruses. In addition, the two amino acid substitution sites are on or near the 150-loop region. Previous studies have suggested that base changes, specifically base extension, in the 150-loop might affect polar amino acids that have stereoscopic clashes with human receptors, thus affecting the receptor binding specificity of H10 and thereby altering receptor preference from α-2,3 (avian-like) to α-2,6 (human-like)-linked sialosides [47, 48]. In this study, the results of the receptor specificity assay suggested that the parental and escape mutant strains of A/chicken/Zhejiang/2CP8/2014 (H10N7) all possessed specificity toward the avian-like receptor and that these mutants did not exhibit altered receptor specificity (Additional file 1).

The two selected mAbs effectively bound their homologous HA antigen in an ELISA experiment and demonstrated significant affinity. Under the same conditions, the 2A9 antibody could effectively bind HA-purified protein at concentrations as low as 2 × 10^–4^ μg/mL, which indicates that it might have superior binding stability. In addition, in the HI experiment, 2A9 had a greater ability to inhibit agglutination of chicken red blood cells in the presence of the H10N7 virus, implying that it has broad specificity against the H10 virus and could prevent the H10N7 virus from attaching to red blood cells. In the mouse experiments, when 2A9 was administered at a dose of 10 mg/kg, the experimental mice gained weight at a faster rate, suggesting that the neutralizing effect of 2A9 in vivo might be more advantageous than that of 1E10. However, the 1E10 antibody was superior to the 2A9 antibody in the neutralization ability of each H10 virus in vitro and exerted significant protective effects when administered for both prophylactic and therapeutic purposes in the H10N7-infected mouse model, with the weight of the mice receiving 1E10 increasing, which further confirmed the consistency of its in vivo protective effect with the results of in vitro cell assays.

In conclusion, we developed two mAbs (1E10 and 2A9) against the HA of H10N7 influenza virus and determined their characteristics and functions. The two mAbs could exert antiviral effects in mice in vivo. Additionally, the regions that bind to the epitope could provide valuable insights for the development of H10 subtype influenza vaccines.

## Supplementary Information

**Additional file 1. Virus receptor binding specificity of parental and escape mutant strains**. Normal chicken red blood cells (containing both α-2,3 and α-2,6 receptors) were prepared, and 1 mL of 10% red blood cells was treated with 1000 IU of α-2,3-specific neuraminidase (NEB, USA) at 37 °C for 1 h to produce sialidase-treated chicken red blood cells, which contained only the α-2,6 receptor. Avian influenza virus A/goose/Zhejiang/112071/2014 (H5N1) and human influenza virus A/Michigan/45/2015 (H1N1) were included in the receptor binding assay as controls. The hemagglutination assay was performed in 96-well plates by incubating 50 µL of twofold serially diluted viruses with 0.5% red blood cells.

## Data Availability

The datasets used and/or analysed during the current study are available from the corresponding author upon reasonable request.
