# Supplementary material for: Neutralizing monoclonal antibodies as effective therapeutics and prophylactics against lethal H10N7 avian influenza infection in a mouse model
Source: Vet Res. 2025 Apr 2;56:75. doi: 10.1186/s13567-025-01504-0 (PMC11966874; doi:10.1186/s13567-025-01504-0)
Supplement: Supplementary file 1 — Additional file 1. Virus receptor binding specificity of parental and escape mutant strains. Normal chicken red blood cells (containing both α-2,3 and α-2,6 receptors) were prepared, and 1 mL of 10% red blood cells was treated with 1000 IU of α-2,3-specific neuraminidase (NEB, USA) at 37 °C for 1 h to produce sialidase-treated chicken red blood cells, which contained only the α-2,6 receptor. Avian influenza virus A/goose/Zhejiang/112071/2014 (H5N1) and human influenza virus A/Michigan/45/2015 (H1N1) were included in the receptor binding assay as controls. The hemagglutination assay was performed in 96-well plates by incubating 50 µL of twofold serially diluted viruses with 0.5% red blood cells. [file 13567_2025_1504_MOESM1_ESM.docx]

**Additional file 1 Virus receptor binding specificity of parental and escape mutant strains.**

| **Virus** | **Receptor binding specificity** | | |
| --- | --- | --- | --- |
|  | **α-2,3** | **α-2,6 and α-2,3** | **α-2,6** |
| A/chicken/Zhejiang/2CP8/2014 (H10N7) | 128 | 128 | < 2 |
| A/chicken/Zhejiang/2CP8/2014 (H10N7)--1E10 | 128 | 128 | < 2 |
| A/chicken/Zhejiang/2CP8/2014 (H10N7)--2A9 | 128 | 128 | < 2 |
| A/goose/Zhejiang/112071/2014 (H5N1) | 256 | 256 | < 2 |
| A/Michigan/45/2015 (H1N1) | < 2 | 128 | 128 |
